# The Advantages and Challenges of Anticancer Dendritic Cell Vaccines and NK Cells in Adoptive Cell Immunotherapy

**DOI:** 10.3390/vaccines9111363

**Published:** 2021-11-19

**Authors:** Elena V. Abakushina, Liubov I. Popova, Andrey A. Zamyatnin, Jens Werner, Nikolay V. Mikhailovsky, Alexandr V. Bazhin

**Affiliations:** 1 Department for Development and Research in Immunology, LLC “Tecon Medical Devices”, 123298 Moscow, Russia; ljubovprokudina@gmail.com (L.I.P.); nickmikh.mrrc@gmail.com (N.V.M.); 2 Belozersky Institute of Physico-Chemical Biology, Lomonosov Moscow State University, 119992 Moscow, Russia; zamyat@belozersky.msu.ru; 3 Institute of Molecular Medicine, Sechenov First Moscow State Medical University, 119991 Moscow, Russia; 4 Department of Biotechnology, Sirius University of Science and Technology, 1 Olympic Ave, 354340 Sochi, Russia; 5 Department of General, Visceral, and Transplant Surgery, Ludwig-Maximilians-University Munich, 81377 Munich, Germany; jens.werner@med.uni-muenchen.de (J.W.); alexandr.bazhin@med.uni-muenchen.de (A.V.B.); 6 German Cancer Consortium (DKTK), Partner Site Munich, 81377 Munich, Germany; 7 Bavarian Cancer Research Center (BZKF), 91054 Erlangen, Germany

**Keywords:** cancer immunotherapy, adoptive cell therapy, cancer vaccines, dendritic cells, tumor antigens, NK cells, NKG2D, cell-mediated immunity

## Abstract

In the last decade, an impressive advance was achieved in adoptive cell therapy (ACT), which has improved therapeutic potential and significant value in promising cancer treatment for patients. The ACT is based on the cell transfer of dendritic cells (DCs) and/or immune effector cells. DCs are often used as vaccine carriers or antigen-presenting cells (APCs) to prime naive T cells ex vivo or in vivo. Cytotoxic T lymphocytes (CTLs) and natural killer (NK) cells are used as major tool effector cells for ACT. Despite the fact that NK cell immunotherapy is highly effective and promising against many cancer types, there are still some limitations, including insignificant infiltration, adverse conditions of the microenvironment, the immunosuppressive cellular populations, and the low cytotoxic activity in solid tumors. To overcome these difficulties, novel methods of NK cell isolation, expansion, and stimulation of cytotoxic activity should be designed. In this review, we discuss the basic characteristics of DC vaccines and NK cells as potential adoptive cell preparations in cancer therapy.

## 1. Introduction

In the 19th century, a young bone surgeon and cancer researcher, William B. Coley, noticed that cancer patients often experienced remission following infections. This observation sparked the idea of cancer immunotherapy. Because of the limited clinical use and toxicity of chemotherapy, immunotherapy may be the best option for increasing the survival of cancer patients [1]. Today, scientists have a number of options for treating cancers via immunotherapy, and the research field is more active than ever. Great strides in immunology and cancer research have led to emerging therapies, which include adoptive cell therapy (ACT), immune checkpoint inhibition, and cancer vaccines—all of which show great promise for treating a multitude of cancers [2].

Although classical cancer therapies are widely used for effective treatment, their therapeutic potential is limited. Due to the novel research in this field, cancer therapy has recently been oriented toward immunotherapy approaches with relatively high safety margins and selectivity compared to chemotherapeutic agents. Optimized adoptively transferred cell-based approaches in combinations with different cancer therapies have the potential to open new roads in cancer immunotherapy. Usually, ACT is used to enhance the function of a patient’s immune system by the injection of activated or modified effector cells, which can kill tumor cells with high specificity. The effects of combined use of ACT with other treatment methods have been described in numerous studies [3,4,5,6,7]. ACT allows the patient’s immune system to be used to destroy cancer cells.

Although the existence of cytolytic effector cells within the tumor can elicit a naturally occurring antitumor response, the functional deficiency of the immune system suggests the necessity of therapeutic intervention to improve the immune function. Tumor response to cell therapy is determined by numerous factors, including the tumor microenvironment (TME) and immunosuppression due to the negative effect of regulatory T cells (Treg) and tumor cells themselves [8]. The TME consists of various immune cells, including B and T lymphocytes, tumor-associated macrophages, DCs, and fibroblasts.

One of the functions of DCs is promoting cooperation between the innate and adaptive immune responses, and this balance can be managed through DC vaccine therapy. Ultimately, the knowledge gained by much research into antigen processing and presentation needs to be translated from bench to bedside. DCs thus have a unique ability to transport tumor antigen to the draining lymph nodes to initiate T cell activation, a process that is required for T cell-dependent immunity [9]. These functions place DCs at the fulcrum of the antitumor T cell response and suggest that regulating the biological activity of these cells is a viable therapeutic approach to indirectly promote an effector T cell response during therapy.

While certain progress has recently been made in treatment with DCs and cytotoxic T lymphocytes (CTLs), the clinical use of CTLs is limited due to the fact that they must meet and recognize tumor antigens to kill tumor cells. However, most of the antigens expressed by tumor cells are closely related or identical to the gene products of normal cells. In contrast, NK cells have antigen-independent cytotoxic activity against transformed cells [10].

NK cells are a prevalent subset of lymphocytes of the innate immune system, and their main function is host defense against pathogens and damaged cells in an MHC-independent manner. The expression of CD56 and/or CD16 and CD3 negative are the main phenotypic characteristics of NK cells [6]. NK cells are cytotoxic against a wide range of tumor cells of solid cancer types in vitro. Antitumor activities of adoptively transferred NK cells in vivo have been demonstrated in preclinical xenograft mouse models of ovarian cancer, glioblastoma, and metastatic colorectal cancer [11,12,13]. The safety of NK cell-based therapy has been confirmed in both autologous and allogeneic haploidentical settings [14,15,16]. The clinical efficacy of this strategy has proven thus far to be limited [13,14,15,16].

The major purpose of cancer immunotherapy is to enhance the activity of immune effector cells by inverting the immunosuppression and/or immune deficiency.

## 2. Dendritic Cells Vaccines

Therapeutic cancer vaccines use cancer-specific antigens to boost the body’s natural defenses for fighting cancer [17]. Scientists prepare many cancer vaccines from patient samples, with the goal of eliciting a potent, long-lasting CD4 plus CD8 T cell expansion, which is necessary for clinical efficacy [18]. Our understanding of T cell antitumor responses has come a long way through incremental steps of elucidating the intricate interaction between cancer cells, T cells, antigen-presenting cells, immunosuppressive cells, and other immune mechanisms [19]. The dynamic interplay between inhibitory and stimulatory signals on T cells modulates the degree of immune activation to allow tolerance to self-antigens (inhibitory) while mounting an adaptive immune response to foreign antigens (stimulatory) [20]. Increasing the effectiveness of all types of cell therapy in oncology is associated with the targeted delivery of the cell product to the site of action, whether it be tumor tissue or lymphoid organs.

Many factors intricately interact with each other within tumor cells and in the TME. Intrinsic factors that cause a decrease in intratumoral T cell infiltration include epigenetic changes and intracellular signaling pathways. However, DCs or other antigen-presenting cells (APCs) are known to play an important role in the infiltration of T cells into the TME. DCs bridge the gap between the innate and adaptive immune response, tipping the direction toward response or peripheral tolerance [21,22]. Moreover, cancer cells affect DCs through different immune escape mechanisms. Manipulating this balance through DC vaccine therapy has therapeutic potential and this is not a novel concept [23]. Early regulatory approvals in some countries include oncophage, a vaccine comprising patient-extracted heat shock protein gp96 for treating kidney cancer, and sipuleucel-T, an immunostimulant vaccine for prostate cancer, which uses patient’s white blood cells after incubation with a prostate cancer antigen and an immune signaling factor [24]. Sipuleucel-T was the first DC therapy approved by the FDA for the treatment of metastatic castration-resistant prostate cancer (mCRPCa).

During mononuclear cell separation from peripheral blood, one of the frequent technical complications is low abundance (0.1–1%) of DCs [25]. Therefore, the density gradient method to enrich the cell product with APCs was used in early research, such as in the development of sipuleucel-T. “Second-generation” DC vaccines use strategies that differentiate monocytes into dendritic-like cells called MoDCs, creating a more readily available source of APCs as monocytes make up ~10% of peripheral blood mononuclear cells (PBMCs) compared to DCs. This method is based on the separation of MoDCs from PBMCs by CD14^+^ receptor through their ability to adhere to plastic overnight culture or by anti-CD14 magnetic microbeads, after which CD14^+^ cells are cultured in elective conditions (addition of cytokines, typically GM-CSF and IL-4 for 4–5 days) to obtain immature DC-like phenotype [26]. In clinical trials, the main parameters affecting efficacy and applicability are immature or mature (including activation agent) status of MoDCs and tumor antigen delivery variation (including protein, peptide, apoptotic tumor cells, lysate from tumor cell lines, or mRNA; Table 1).

In melanoma, whilst only a small proportion, i.e., 4%, of intradermally injected DCs, migrate to local lymph nodes, those that do activate CD8^+^ T cells in this model, thus overcoming the TME [27]. There have been several clinical trials in prostate cancer patients with MoDCs (Table 1). Vaccination is a developing field, with various anti-cancer vaccines in clinical trials.

## 3. Interaction of DCs and NK Cells

However, the clinical efficacy and application of DCs for immunotherapy are limited by the impossibility of assessing their migration ability after adoptive transfer, since these cells do not reach their therapeutic localizations in all patients. It is also difficult to trace their in vivo activity and temporal distribution in the body, which requires additional research.

Within the TME, NK cells produce a set of chemokines, namely CCL4, CCL5, XCL1, and XCL2, to recruit conventional type 1 DCs (cDC1) and also synthesize factor FLT3L, stimulating cDC1 recruitment and differentiation [28,29,30]. The NK cell–cDC1 cross-talk promotes migration patterns of both cell types and regulates the interaction between them [28]. NK cells perform so-called “editing” of DCs, during which activated NK cells eliminate immature DCs, and therefore bona fide tolerogenic DCs, while sparing activated/mature DCs able to efficiently induce the subsequent adaptive immune response in secondary lymphoid organs. Under the influence of cytokines (IL-2, IL-12, or IL-18) released by mature DCs, NK cells produce IFN-γ, TNF-α, or GM-CSF, guiding DC maturation [31,32]. Besides cytokine secretion, NK cells influence the maturation of DCs via the ligation of CD40/CD40L [33].

Upon exposure to tumor cells or the additional inflammatory signals, IL-18-primed NK cells attract immature DCs with CCL3 and CCL4, inducing cDC1 production of CXCL9, CXCL10, and CCL5 [34]. These chemokines attract expanded cytotoxic T cells to the tumor site, providing antitumor activity [35]. In addition to cytokines, DCs modulate NK cell activity via interaction with adhesion molecules, such as CD155 and CD112, with NK cell receptors DNAM-1 (activation), TIGIT (inhibition), and CD96 (inhibition) [36,37,38,39]. Detailed study of the bidirectional relationship between NK cells and DCs in TME by which the immunosuppressive mechanism is antagonized should continue.

It was shown when used DCs in combination with cytokine-induced killer (CIK) cells, immunotherapy improved the survival of patients diagnosed with stomach cancer and colorectal carcinoma [40]. The one-year survival rates were 58.1% (230/396) and 76.5% (202/264) in the control group and the group subjected to combined therapy involving immunotherapy, respectively.

## 4. Characteristics of NK Cells

The antitumor activity of NK cells is the subject of intensive research in the field of cancer immunotherapy [41]. NK cells are part of the innate immune system with unique advantages, including the potential for “off-the-shelf” therapy. NK cells can eliminate target cells controlled by signals derived from activating and inhibitory receptors [42]. The emergence of a cytotoxic NK cell response depends on the combination of various surface receptors. Many of them belong to the immunoglobulin-like receptor (KIR) family or lectin-like receptors. Either can inhibit or activate NK cells [43]. In particular, the germline-encoded NK receptors include the activating receptors for the elimination of tumor cells, namely NKG2D, DNAM-1, NKp80, CD2, and Tim-3; the natural killing receptors NKp30, NKp44, and NKp46; the signaling lymphocyte activating molecule (SLAM) family (2B4, NTB-A, and CRACC); and MHC-dependent KIRs (KIR2DS, KIR3DS) and CD94/NKG2C. Among main inhibitory receptors identifying MHC class I molecules are KIR2DL and KIR3DL; CD94/NKG2A and LIRs; and non-MHC ligand receptors such as CD96 and TIGIT [44,45]. Normal host cells are protected from NK cells attacks through inhibitory KIRs that identify the self-MHC class I molecules [45].

The interaction between inhibitory KIRs and self-MHC molecules, a process termed “licensing”, governs the maturation of NK cells, which become competent to be further triggered through activation receptors [46]. Except for MHC downregulation, NK cell receptors can be unregulated by binding molecules that are overexpressed on cancer cells. As an example, such ligands for the activating NKG2D receptor are MHC I polypeptide-related sequence A (MICA), MICB, and others, expressed by cancer cells mainly in response to cellular stress [47]. A separate mechanism known as antibody-dependent cell cytotoxicity (ADCC) results in the elimination of antibody-coated cells via the CD16 FcRIII receptor [48].

With antiviral, anti–graft-versus-host disease, and anticancer potential, adoptive immunotherapy with NK cells has emerged as a promising therapeutic [4,13]. Based on the unique properties of NK cells, the therapeutic use of autologous or allogeneic NK cells may be of pivotal and clinical significance in human cancer immunotherapy [4,41]. The NK cell-based immunotherapy is potentially safer than other cell-based immunotherapies because the NK cells do not trigger a cytokine storm, as is seen sometimes in CAR-T cell therapy, which often is used for blood cancers, nor do the NK cells cause graft-versus-host disease, which sometimes follows a stem cell transplant [13].

In cancer patients, NK cell function is generally inhibited due to the reduced expression of NK cell-activating receptors, thus impairing their tumor-killing activity [49,50]. For the rational use of NK cells in anticancer therapy, it is necessary to know the morphological features and functional characteristics of these innate immunity lymphocytes [51].

Purified NK cells had demonstrated evidence of therapeutic potential for a wide variety of human malignancies, including sarcomas [52], myeloma [53], carcinomas [54,55,56], lymphomas [57], and leukemia [1,58]. However, the clinical efficacy and application of NK cell immunotherapy have been limited by the inability to obtain sufficient cell numbers for adoptive transfer, as these cells represent a small fraction of peripheral white blood cells, expand poorly ex vivo, and have limited life spans in vivo.

The majority of NK-based immunotherapy strategies are still at the early stages of clinical trials (phase I and II) for use in cancer treatment. Table 2 summarizes updated clinical trials of NK cell-based therapy for all solid tumors.

Several clinical studies report that NK cell-based therapy is a promising method of cancer treatment [40,43]. However, progress in immunotherapy of cancer with NK cells has been insignificant, because it was not possible to obtain a sufficient number of NK cells to adequately assess the effectiveness in preclinical and clinical trials [59]. Currently, some positive clinical results have been achieved in this direction, including the use of cytokines and feeder cells to activate NK cells ex vivo, adoptive transfer of NK cells with antitumor activity, or genetically modified CAR lymphocytes [51]. Therefore, there is considerable interest in the development of ex vivo methods for generating NK cells.

## 5. Generation NK Cells Ex Vivo

### 5.1. Methods of NK Cell-Based Immunotherapy

One of the well-known methods of NK cell-based adoptive immunotherapy involves ex vivo expansion and activation [60]. This method has been developed to increase both the number and antitumor activity of NK cells to overcome the immunosuppression that is commonly observed in solid tumors.

Various sources of NK cells are currently used for ACT; these include autologous NK cells, allogeneic NK cells, NK cell lines, stem cells, and genetically engineered NK cells.

Cytotoxic NK lymphocytes can be obtained from PBMCs and CD34+ hematopoietic stem cells. In addition, they can be derived from cord blood stem cells or induced pluripotent cells, which have a longer culture period (more than 3 weeks) [41,61]. Studies with native autologous NK cells have yielded disappointing results. To activate the NK cells, obtained from healthy donors’ PBMCs, different combinations of cytokines are used, mainly with IL-2. However, infusion of cell preparations stimulated with IL-2 led to the development of severe side effects, such as vascular leak syndrome and liver toxicity [62].

Several groups of researchers use NK cell lines (NK-92, NKL, KYHG-1, YT, NKG) for therapy. As an alternative to primary cells, the Food and Drug Administration (FDA)-approved NK cell line NK-92, which can be expanded to high numbers under good manufacturing practice conditions, can be used. 

Genetically modified NK cells with mutations in cytokine pathways or with chimeric antigen receptors can be used [63], although further research is necessary to identify their in vivo activity and distribution in the body. Apheresis and removal of T and B lymphocytes is an approved and GMP-compliant method for obtaining a pure subpopulation of NK cells [64]. This approach has several drawbacks, including lengthy procedure. Moreover, due to the reduction in lymphocyte amount, autoimmune diseases and immunodeficiency can develop. In cancer patients, due to the toxic side effects of chemotherapy, myelosuppression is possible, which is manifested by lymphopenia, and suppression of the cellular link of immunity and apheresis will only exacerbate the situation.

Therefore, most often NK cells are derived from the peripheral blood of patients or donors. Circulating mature NK cells can be divided into two main subsets: minor regulatory CD56bright/CD16low NKs, which secrete high amounts of proinflammatory cytokines and are the most abundant in secondary lymphoid organs, and major cytotoxic CD56dim NKs, representing about 90% of circulating NK cells, which during several maturation steps downregulate CD56 and upregulate CD16 [65,66]. The same result can be achieved by ex vivo cultivation of NK cells. When activated on NK cells, there is an increase in the expression of receptors, including nonspecific HLA receptors such as NKp30, NKp44, NKp46, NKG2D, DNAM-1, NKp80, CD59, NTB-A, and 2B4 [44,67,68,69].

However, it seems difficult to compare different protocols for culturing NK cells since they are extremely heterogeneous. The ex vivo activation period of NK cell cultivation can vary from several hours to weeks. Numerous initial components of media with different proportions of NK cells and combinations of several cytokines at different concentrations are used. In vitro generation of PBMCs in different combinations with cytokines, antibodies, growth factors, or feeder cells remains the most generally accepted method for NK cell and CTL activation [70,71]. As for the production of NK cells in vitro, they use, in particular, the cytokines IL-2, IL-15, IL-18, and IL-21 and genetically modified feeder cells that increase the cytotoxicity of NK cells [41,72]. These methods allowed the number of NK cells ex vivo to be increased by about 35–1000 times in 12–20 days [53], with one exception: the number of generated cells obtained from hematopoietic stem cells and umbilical cord blood cells increased by more than 15,000 times in 35 days [73].

The common γ-chain cytokines IL-2, IL-15, and IL-21 have all been extensively studied with regard to NK cell activation, maturation, and proliferation. IL-15 has a well-recognized role in maturation, survival, and homeostatic expansion of NK cells, which supported its application in developing IL15-expressing feeder cells (mbIL15). The discovery of IL-21 was linked to a role in promoting proliferation and maturation of NK cells [74]. IL-21 synergizes with IL-2, IL-15, and Flt-3L in the generation of NK cells from bone marrow and cord blood [74,75]. Although all three cytokines signal through the JAK/STAT pathway, IL-2 signals mostly through STAT5 with some STAT3 and STAT5 activation, IL-15 signals almost exclusively through STAT5, and IL-21 signals primarily through STAT3 with very little STAT1 and STAT5 involvement [76].

While the majority of investigations of ex vivo NK priming strategies rely on one or more cytokines, some studies indicate that cytokine supplement is not critical for NK cell priming [64]. To investigate the potential of these cytokines in supporting NK cell propagation, researchers used a variety of K562-based feeder cells expressing membrane-bound chimeras of IL-21 (mbIL21) and IL-15 and investigated NK cell expansion, phenotype, and function in response to repeated weekly stimulation with irradiated feeder cells. The generation of NK cells under these conditions and a manifold increase in their number were ensured with prolonged cultivation for at least 21 days [41]. One of the most efficient NK cell expansions was observed in the protocol with modified mbIL15 K562 cells and 4–1BBL [64]. One leucocyte apheresis product was yielded in the huge NK cell product, which was enough for at least four infusions at 50 million cells/per kg. It has been shown that ex vivo cultivation of NK cells can act on tumor cells resistant to the function of native NK cells [60]. It was proposed that the cytokine preactivated NK cells were “memory-like” with an enhanced response to cytokine or activating receptor restimulation weeks or months after the initial preactivation [77].

Methods for culturing NK cells with or without feeder cells are being studied. All this requires the study and selection of the optimal conditions for the generation of NK cells.

### 5.2. Interaction between NKG2D Receptor and Its Ligands for NK Cell Therapy

An innovative approach in cancer immunotherapy is ACT. Genetically engineered immune cells, including T cells, NK cells, γδ-T cells, NKT cells, and even macrophages to express antigen-specific TCRs or CARs are of deep interest for researchers working on progress in cellular immunotherapies [55,78,79].

Several preclinical studies confirmed the effectiveness of CAR-NK cell therapy against hematological malignancies in both in vivo and in vitro experiments [80].

CAR-NK cell immunotherapy compared to the CAR-T cells has been shown to have advantages in safety (concerning reduced risk for graft-versus-host disease (GVHD), cytokine release syndrome (CRS), and neurotoxicity), in cytotoxic activity mechanisms (CAR-dependent and NK cell receptor-dependent mechanisms), and in “off-the-shelf” manufacturing (CAR-NK cells can be generated from multiple sources, including NK92 cell lines, PBMCs, umbilical cord blood (UCB), and induced pluripotent stem cells (iPSCs), eliminating the need for a patient-specific product) [79,81,82].

Among activating NK cell receptors, NKG2D, which recognizes stress-induced ligands MICA, MICB, and ULBP1-6 expressed on tumor cells, is one of the best-studied, but there are still some questions about the mechanisms of the impact of NKG2D ligands on NK cell function [83]. NKG2D ligand release can occur by shedding, and these soluble ligands prevent NK cell–tumor cell interaction and the cytotoxic response [84,85]. Apart from their genetic diversity, NKG2D ligands are characterized by the presence of membrane-bound (m) and soluble isoform (s) and by the propensity to elicit antibody-mediated allogeneicity [86]. Dhar et al. (2021) demonstrated the molecular mechanisms of these interactions. The interaction of the sMIC and the NKG2D selectively activates the CBM signalosome (via the CARMA1–BCL10–MALT1 pathways) and stimulates NK cells to secrete protumorigenic cytokines (triggering the NF-κB cascade) [87]. Conversely, the interaction of mMIC with the receptor activates the PLCγ2/Vav1/SLP-76 and ERK/JNK pathways, triggering the cytotoxic effector function of NK cells (also enhancing IFNγ secretion). In murine models (mouse splenic NK cells cultured with prostate tumor cells TRAMP-C2), it was observed that the addition of sMIC-binding mAb B10G5 upon cell stimulation with recombinant sMICA did not lead to activation of proinflammatory signaling pathways [88]. As a result, it was hypothesized that antibodies binding soluble forms of sMIC ligands could be used to improve NK cell therapy in the treatment of MIC-positive tumors. This idea was confirmed in an experiment on NSG mice inoculated with human MIC-positive PL12 pancreatic cancer cells, which received adoptive therapy in the form of NK92 cells. For a group of mice that received a combination therapy of NK92 cells and mAb B10G5, 100% survival was demonstrated [87].

Fuertes et al. (2021) also discussed the application of monoclonal antibodies in adoptive therapy depending on their ability to induce ADCC [83]. Among the mAb ligands, MICA was chosen as a target since it is most often expressed by tumor cells.

The observation of melanoma patients receiving ipilimumab (anti-CTLA4 mAb) and autologous tumor cells engineered to produce GM-CSF as therapy revealed that due to the activation of NKG2D expression on NK cells and cytotoxic lymphocytes, some patients spontaneously developed antibodies against MICA, which bound sMICA from plasma and promoted opsonization of tumor cells for dendritic cell cross-presentation [89].

As another way to induce anti-MICA antibodies to be used as antitumor therapy, a chimeric protein (BLS-MICA) was developed, consisting of the MICA ectodomain fused with a lumazine synthase from *Brucella* spp. (BLS). Immunization of mice with BLS-MICA led to the generation of high titers of anti-MICA antibodies, which during in vitro experiments neutralized the MICA ligand expressed on the cell surface of human tumor cells. The therapeutic effect of immunization with BLS-MICA resulted in significant inhibition of the growth of MICA-expressing tumors by binding excess sMICA molecules, tumor elimination by ADCC, and prevention of tumor escape [90].

Among CAR-NK cell technologies targeting NKG2D ligands, two drugs are currently undergoing clinical trials: NCT03415100, the treatment of metastatic tumors with autologous and allogeneic NK cells, and NCT04623944, the treatment of acute myeloid leukemia with NKX101 (allogeneic CAR-NK cells); according to both clinical trials, there have been no published results yet [91,92].

During the coronavirus pandemic, it has also been suggested to use CAR-NK cell therapy to treat COVID-19 [93]. The NKG2D receptor may be involved in the recognition of virus-infected cells. IL15 superagonist and GM-CSF neutralizing scFv-secreting NKG2D-ACE2 from cord blood CAR-NK cells were constructed to be used in therapy. IL15 superagonist can increase the duration of drug circulation in the blood and activate the cytotoxicity of NK cells. Neutralization of GM-CSF during CAR-cell therapy leads to the elimination of neurotoxicity and the development of a “cytokine storm”. The ACE2 receptor recognizes the SARS-CoV2 S-protein. Phase I/II clinical trials of this drug have started (NCT04324996) [94].

## 6. Conclusions and Future Research

Although the application of ACT in the therapeutic treatment of different cancers has high evaluations, specific research is required to enhance the efficiency, especially in the treatment of solid tumors [95]. Additional attention should be focused to improve brief persistence and poor migration of immune cells within the tumor microenvironment, anergy, and weak proliferation of immune cells. More research should be directed towards exploring more effective and personalized strategies to improve the performance of ACT, especially considering immunosuppressive mechanisms of solid tumors.

In summary, NK cells can be artificially activated to be used as a highly functionalized anticancer tool against hematopoietic malignancies and, dependent on successful further rearming and mobilization, against solid tumors in the future [5,80,82,96]. The difficulty in obtaining large quantities of NK cells, expanding to clinical scale ex vivo, and sustaining in vivo survival and activity of infused NK cells has encumbered the progress [97,98,99].

Dendritic cells are also emerging as critical regulators of the immune response within tumors. Understanding how to augment the function of these intratumoral DCs could offer new approaches to enhance immunotherapy, in addition to improving the cytotoxic and targeted therapies that are partially dependent upon a robust immune response for their efficacy. DCs could play a pivotal role in triggering the antitumor efficacy of CIKs. The combination of CIK cells and DC vaccination may have a major impact on immunotherapeutic protocols for patients with cancer, especially with solid tumors. One of the possible mechanisms is DC activation by the cytotoxicity of CIK cells leading to the upregulation of the synthesis of cytokines involved in the anticancer effect. ACT can simultaneously improve the antitumor immune response. Specifically, DC-CIK cells can increase T lymphocyte subsets, CIK cells, NK cells, and immunoglobulins in peripheral blood to enhance antitumor immunity [100]. DC-CIK cell immunotherapy of patients without recurrences resulted in prolonged survival time and strengthened immune response with no specific side effects and is promising for future therapeutic trials [40,101].

The main objective of modern immunotherapy is, on the one hand, stimulating the antitumor immune response and, on the other hand, disabling suppressor mechanisms that support tumor growth. Such therapy induces and stimulates the antitumor immune response of a cancer patient; the immunotropic effects prolong cancer remission periods in patients and improve their quality of life. It is believed that ACT, alone or in combination with other therapies, may hold great promise for the treatment of cancers.

## Figures and Tables

**Table 1:** Selected clinical trials of dendritic cell-based cancer vaccines.

Trial ID	Enrollment	Condition	Interventions	Trial Phase	Date
NCT00703105	36	Ovarian cancer	Ontak (anti-CD25), DC ^1^ vaccine + ontak	II	2008–2018
NCT01204684	60	Glioma, astrocytoma, astro-oligodendroglioma, glioblastoma	AT ^2^ TL ^3^-pulsed DC + 0.2% resiquimod, DC vaccination + polyICLC ^4^	II	2010–2018
NCT01803152	56	Sarcoma, soft tissue sarcoma, bone sarcoma	DC vaccine, TL, gemcitabine, imiquimod, leukapheresis	I	2014–2019
NCT01885702	25	Colorectal cancer	Neoantigen-loaded DC vaccination	I/II	2010–2016
NCT01946373	10	Melanoma	Cyclophosphamide, fludarabine, T cells, interleukin-2, DC vaccine	I	2013–2018
NCT01957956	21	Newly diagnosed glioblastoma	TL-pulsed AT DC vaccine + temozolomide	I	2013–2016
NCT01983748	200	Uveal melanoma	AT DC loaded with AT tumor RNA	III	2014–2022
NCT02301611	120	Melanoma	AT TL + YCWP ^5^ + TLPLDC ^6^ Vaccine, placebo	II	2015–2019
NCT02496520	10	Advanced solid tumors, sarcoma, central nervous system tumor	DC, surgery as needed, chemotherapy as needed, radiation therapy as needed	I/II	2014–2018
NCT02503150	480	Metastatic colorectal cancer	Antigen pulsed DC + chemotherapy, chemotherapy	III	2015–2019
NCT02678741	45	Metastatic melanoma	TLPLDC vaccine in addition to ICPI ^7^ of choice	I/II	2016–2019
NCT02718391	120	Melanoma	DC pulsed with autologous TL	II	2015–2019
NCT02775292	12	Adult solid neoplasm, childhood solid neoplasm, metastatic neoplasm	Nivolumab, NY-ESO-1 reactive TCR ^8^ retroviral vector transduced AT PBL ^9^, NY-ESO-1(157-165) peptide-pulsed AT DC vaccine	I	2017–2019
NCT03014804	30	Recurrent glioblastoma	AT DC pulsed with TL, nivolumab	II	2018–2020
NCT03300843	86	Melanoma, gastrointestinal, breast, ovarian, pancreatic cancer	DC vaccine loaded with neoantigen coding peptide	II	2018–2027
NCT03360708	20	Recurrent glioblastoma	CIK ^10^ cells, TL-pulsed AT DC vaccine	I	2018–2022
NCT03395587	136	Newly diagnosed glioblastoma	AT DC pulsed with AT TL	II	2018–2022

^1^ DC—dendritic cell; ^2^ AT—autologous; ^3^ TL—tumor lysate; ^4^ ICLC—interstitial Cajal-like cell; ^5^ YCWP—yeast cell wall particles; ^6^ TLPLDC—tumor lysate, particle-loaded, dendritic cell; ^7^ ICPI—immune checkpoint inhibitors; ^8^ TCR—T cell receptor; ^9^ PBL—peripheral blood lymphocyte; ^10^ CIK—cytokine-induced killer.

**Table 2:** Selected clinical trials of NK cell-based immunotherapies.

Trial ID	Enrollment	Condition	Interventions	Trial Phase	Date
NCT01212341	18	Malignant lymphomas, solid tumors	Allogeneic NK ^1^ cells	I	2010–2013
NCT02030561	29	Breast, gastric cancer	Autologous NK cells + trastuzumab	I/II	2014–2018
NCT02100891	15	Neuroblastoma, rhabdomyosarcoma	Allogeneic NK cells with HLA ^2^-HCT ^3^	II	2014–2021
NCT02118415	90	NSCLC ^4^ Stage IIIA/B	Hsp70-peptide TKD/IL-2 activated, autologous NK cells	II	2014–2019
NCT02839954	10	Hepatocellular carcinoma, non-small-cell lung cancer, pancreatic carcinoma	Allogeneic anti-MUC1 CAR ^5^-pNK cells	I/II	2016–2018
NCT02843126	30	Breast cancer recurrent	Trastuzumab combined with NK cells	I/II	2016–2019
NCT02843581	60	Metastatic esophageal cancer	Cryosurgery combined with NK cells	I/II	2016–2019
NCT02843607	30	Metastatic renal cell cancer	Cryosurgery combined with NK	I/II	2016–2019
NCT02843815	30	Non-small-cell lung cancer metastatic	Cryosurgery combined with allogeneic NK cells	I/II	2016–2019
NCT02844335	60	Breast cancer recurrent	Cryosurgery combined with NK cells	I/II	2016–2019
NCT02845856	30	Recurrent non-small-cell lung cancer	Cetuximab combined with NK cells	I/II	2016–2019
NCT02845999	9	Gastrointestinal metastatic cancer	Allogeneic NK cells with cetuximab	I	2009–2013
NCT02849314	30	Recurrent laryngeal cancer	Cryosurgery combined with NK cells	I/II	2016–2019
NCT02849327	30	Pharyngeal cancer	Cryosurgery combined with NK cells	I/II	2016–2019
NCT02849353	30	Recurrent ovarian cancer	Cryosurgery combined with NK cells	I/II	2016–2019
NCT02849379	30	Recurrent tongue cancer	Cryosurgery combined with NK cells	I/II	2016–2019
NCT03358849	9	Advanced biliary tract cancer	Allogeneic NK Cell (“SMT-NK”)	I	2017–2018
NCT03410368	120	Small cell lung cancer	Autologous NK cells	II	2018–2020
NCT03415100	30	Solid tumors	CAR-NK cells targeting NKG2D ligands	I	2018–2019
NCT03656705	5	Non-small-cell lung cancer	CCCR ^6^-modified NK92 cells	I	2018–2022
NCT03662477	10	Advanced lung adenocarcinoma	Autologous NK cells	I	2018–2021
NCT03882840	30	Cancer lack of MHC ^7^-I expression	Autologous-induced T cell-like NK cells	I/II	2019–2022
NCT03931720	20	Malignant tumor	ROBO1 specific BiCAR-NK/T cells	I/II	2019–2022
NCT03941457	9	Pancreatic cancer	ROBO1 CAR-NK cells	I/II	2019–2022
NCT04324996	90	COVID-19	NK cells, IL15-NK cells, NKG2D CAR-NK cells, ACE2 ^8^ CAR-NK cells, NKG2D-ACE2 CAR-NK cells	I/II	2020–2022
NCT04385641	18	Gastric cancer	Allogeneic UCB-NK cells	N/A	2019–2021
NCT04623944	64	AML ^9^	CAR NK cells	I	2020–2038

^1^ NK—natural killer; ^2^ HLA—human leukocyte antigen; ^3^ HCT—haploidentical hematopoietic cell transplantation; ^4^ NSCLC—non-small-cell lung cancer; ^5^ CAR—chimeric antigen receptor; ^6^ CCCR— chimeric costimulatory converting receptor; ^7^ MHC—major histocompatibility complex; ^8^ ACE2—angiotensin-converting enzyme 2; ^9^ AML—acute myeloid leukemia.

## Data Availability

All the information and data referred to in this review are included in the original publications.
